# Norepinephrine affects the interaction of adherent-invasive *Escherichia coli* with intestinal epithelial cells

**DOI:** 10.1080/21505594.2021.1882780

**Published:** 2021-02-04

**Authors:** Sobieszczańska Beata, Turniak Michał, Olbromski Mateusz, Walczuk Urszula, Marcin Choroszy, Tukiendorf Andrzej, Dzięgiel Piotr

**Affiliations:** a Department of Microbiology, Wroclaw Medical University, Wroclaw, Poland; b Department of Histology and Embryology, Wroclaw Medical University, Wroclaw, Poland; c Department of Public Health, Wroclaw Medical University, Wroclaw, Poland

**Keywords:** Norepinephrine, AIEC, adherence, invasion, CEACAM6

## Abstract

Norepinephrine (NE), the stress hormone, stimulates many bacterial species’ growth and virulence, including *Escherichia coli*. However, the hormone’s impact on the adherent-invasive *E. coli* (AIEC) implicated in Crohn’s disease is poorly understood. In the study, we have investigated the effect of NE on the interaction of six AIEC strains isolated from an intestinal biopsy from 6 children with Crohn’s disease with Caco-2 cells. Our study focused on type 1 fimbria and CEACAM6 molecules serving as docking sites for these adhesins. The study results demonstrated that the hormone significantly increased the adherence and invasion of AIEC to Caco-2 cells in vitro. However, the effect was not associated with the impact of NE on the increased proliferation rate of AIEC or the *fimA* gene expression vital for their interaction with intestinal epithelial cells. Instead, the carcinoembryonic antigen-related cell-adhesion-molecule-6 (CEACAM6) level was increased significantly in NE-treated Caco-2 cells infected with AIEC in contrast to control uninfected NE-treated cells. These results indicated that NE influenced the interaction of AIEC with intestinal epithelium by increasing the level of CEACAM6 in epithelial cells, strengthening their adherence and invasion.

## Introduction

Adherent-invasive *Escherichia coli* (AIEC) is a pathobiont implicated in Crohn’s disease. AIEC characterizes the ability to adhere to and invade human intestinal epithelial cells [1]. Another essential characteristic of this group of *E. coli* strains is their ability to survive and replicate within macrophages without inducing their death [2]. AIEC interacts with the carcinoembryonic antigen-related cell-adhesion-molecule-6 (CEACAM6) at the apical surface of epithelial cells via type 1 pili that promote their adherence and invasion of intestinal epithelium [3,4]. Flagella are another crucial virulence factor promoting adherence and invasion of AIEC into epithelial cells and stimulating the secretion of interleukin-8 [5]. Other virulence factors described in AIEC include long polar fimbria, conferring bacterial interaction with Payer’s patches [6], and the outer membrane protein A (OmpA) that interacts with the endoplasmatic-reticulum-stress-response glycoprotein Gp96 on the intestinal epithelial cells, promoting bacterial invasion [7].

The frequency of AIEC isolation from patients with Crohn’s disease ranges from 21% to 63%, depending on the isolation and sample size methods. AIEC strains are isolated from a small percentage of healthy individuals, which suggests that their involvement in Crohn’s disease is associated with individual factors inclining to develop the disease [8]. Multiple genetic polymorphisms predisposing to Crohn’s disease’s development appear to be an essential host factor promoting AIEC interaction with the intestinal epithelium. Mutations in the cytoplasmic nucleotide-binding oligomerization domain 2 (NOD2) in CD patients result in an increased immune response to bacterial antigens [9].

Overexpression of CEACAM6 receptors at the apical surface of intestinal epithelial cells in patients with CD facilitates AIEC adherence. Additionally, dysfunctional regulation of tight junction proteins associated with CEACAM6 overexpression promotes AIEC invasion into the intestinal mucosa. Decreased protective meprins, proteases degrading bacterial type 1 pili, observed in CD patients, further increase AIEC colonization of intestinal epithelium. Another significant factor favoring successful colonization of AIEC is gut dysbiosis and inflammation of intestinal mucosa that predisposes to the overgrowth and expansion of AIEC in these patients [9,10].

Innervation of Peyer’s patches of the intestinal mucosa with sympathetic cholinergic nerves leads to catecholamines’ (norepinephrine, epinephrine, and dopamine) release upon stimulation, e.g., via uptake of bacteria [11]. At high concentrations, these neuroendocrine hormones increase bowel peristalsis, modulate immunity, and interfere with human body homeostasis, as well as the outcome of infections caused by intestinal pathogens [12]. Nearly half of the norepinephrine synthesized in the human body is produced and utilized within the enteric nervous system influencing both, the host organism and intestinal microbiota [13,14]. Catecholamines influence microorganisms in at least three different ways. They facilitate the acquisition of iron from host iron-binding proteins, i.e., transferrin and lactoferrin, and act as signaling molecules that activate bacterial adrenergic-like QseC receptors [8,11,15]. The effect of NE on the gut microbiome is increased microbial growth rate and enhanced expression of virulence factors through a quorum sensing mechanism [16,17]. Moreover, these hormones modulate the interaction of microbiota with intestinal epithelium via adrenergic signaling. The ex vivo study on porcine colon explants rich in Peyer’s patches evidenced that NE increased the uptake of enterohemorrhagic *E. coli* (EHEC) O157:H7 and *Salmonella enterica* serovar Typhimurium [18,19]. Brown et al. [18] have demonstrated that a neuronal conduction blockade in Peyer’s patch explants with saxitoxin, a neuronal toxin, significantly decreased *Salmonella* Typhimurium uptake. In turn, Green et al. [19] have proven that NE via α2-adrenergic receptors increases EHEC adherence to the colonic mucosa. These studies confirmed the crucial role of adrenergic signaling in the NE-induced colonization of the intestinal epithelium by pathogens and indicated the complex nature of these interactions.

The effect of NE on the interaction of adherent-invasive *E. coli*, as a distinct group of pathogenic *E. coli*, with intestinal epithelium is poorly understood, laying the groundwork for our research. In the study, we determined the adhesion and invasion of AIEC to Caco-2 cells in the presence of norepinephrine, followed by this hormone’s effect on the expression of selected factors, like type 1 fimbria and CEACAM6 molecule that could influence the interaction of AIEC with intestinal epithelial cells.

## Materials and methods

### E. coli *strains and culture media*

A six *E. coli* strains isolated from biopsy specimens of 6 children (mean age 11.1 years, ranging from 7 to 18 years) with Crohn’s disease, diagnosed in the Department and Clinic of Pediatrics and Gastroenterology of the University of Medicine, Wroclaw, Poland, were examined in the study. All these strains were confirmed as *E. coli* by their biochemical characteristics using an Enterotest assay. Based on the ability to adhere to and invade intestinal epithelial cells, as well as to survive within macrophages, all these strains were recognized as AIEC (Supplementary Materials; Table 1). A prototype AIEC LF82 (O83: H1) strain, kindly provided by Dr. Arlette Darfeuille-Michaud, Université d’Auvergne, France, was included in the study as a positive control. *E. coli* were routinely cultured overnight in Luria broth (LB) with shaking at 37°C, and then transferred into a growth-restricting SAPI-serum medium (6.25 mM NH^4^NO^3^, 1.84 mM KH^2^PO^4^, 3.35 mM KCl, 1.01 mM MgSO^4^ and 2.77 mM glucose, pH 7.5), supplemented with non-inactivated 30% (v/v) bovine serum (FBS), and with 50 µM of L-norepinephrine bitartrate (SAPI-serum-NE) in phosphate-buffered saline (pH 7.4), 0.2 µm filter sterilized. NE concentration used in the study has been chosen based on a previous report [20].

#### PCR and RT-qPCR assays

The presence of the *fimA* gene AIEC strains was confirmed in PCR reaction using primers presented in Table S2 (Supplementary Materials). The expression of *fimA* by AIEC was investigated using quantitative real-time PCR. The qRT-PCR reactions were run with mRNA of AIEC cultured in SAPI-serum medium for 24 h, 24 h cultures in SAPI-serum medium subsequently subcultured in MEM medium for 3 h, and 24 h cultures in SAPI-serum medium subsequently subcultured in MEM medium with 50 µM NE (MEM-NE) for 3 h. RNA was extracted using the RNeasy Mini Kit and transcribed to cDNA with the iScriptTM. Reverse Transcription Supermix for RT-qPCR. The reaction was carried out in 10 μl volumes using the Sso AdvancedTM Universal SYBR® Green Supermix on a MIC Real-Time PCR System. The specific primers for *fimA* and *dnaE*, and *rpoS* housekeeping genes are presented in Table S2 (Supplementary Materials). RT-PCR reactions have run in triplicate in the following conditions: activation of the polymerase at 95°C for 15 min, initial denaturation at 95°C for 15 sec, annealing at 60°C for 20 sec, and elongation at 72°C for 20 sec followed by 45 cycles. The expression of CEACAM6 in epithelial cells was determined in Caco-2 cells infected with AIEC strains for 3 hours. RNA was isolated using the Aurum TM Total RNA kit (Bio-Rad) according to the manufacturer’s instruction. RNA concentration was quantified using a NanoDrop ND-1000 spectrophotometer, and the 260/280 and 260/230 ratios were examined for protein and solvent contamination. The relative mRNA expression of the genes examined was normalized against the reference genes *Rps-11*, α-tubulin and β-actin, calculated with the ∆∆Ct method.

#### Cell line

Caco-2 cell line (ATCC HTB37TM) was maintained in a minimal essential medium (MEM) supplemented with 10% of fetal bovine serum (FBS), 1 M sodium pyruvate, 1 M nonessential amino acids (NEAA), 100 U/ml penicillin, and 100 µg/ml streptomycin, at 37°C in a humid atmosphere with 5% CO_2_. Cells were routinely screened for mycoplasma contamination using Hoechst staining. For experimental analyses, Caco-2 cells were seeded at 5 × 10^4^ cells per well in 24-well culture plates and cultured ten to eleven days to a confluent monolayer. In an in vitro adherence and invasion assays, Caco-2 cells were untreated or treated for 3 hours with 50 µM NE added to the cell-culture medium 10 min before infection with AIEC.

#### Adherence and internalization assays

Overnight AIEC cultures in SAPI-serum medium were harvested and suspended in saline to the optical density 6 × 10^8^ CFU/ml established spectrophotometrically at 600 nm and used to infect Caco-2 cells at a multiplicity of infection (MOI) of 50 bacteria per cell. At 3 hours post-infection, cells were washed three times with PBS and lysed with 0.1% Triton X-100. Serial dilutions of bacterial lysates were plated onto nutrient agar and incubated overnight at 37°C to count bacterial colonies (CFU). The invasion assay was performed in the same manner as the adherence assay with an additional 1 hour of incubation of Caco-2 cells in MEM medium containing gentamycin (100 µg/mL) to kill extracellular bacteria. Three separate experiments were performed in triplicate in three independent experiments for adherence and invasion assays.

#### Growth responsiveness of AIEC to norepinephrine

The impact of NE on the growth of AIEC was investigated using stationary cultures in LB medium that were inoculated into SAPI-serum medium supplemented with 50 µM NE to achieve a density of 1 × 10^2^ CFU (colony-forming units) per ml. Cultures were incubated statically at 37°C in a humidified atmosphere with 5% CO_2_ for 24 hours. The density of AIEC cultures was measured at two-time points, t = 0 and t = 24 h, by enumeration of CFU on nutrient-agar plates with a standard dilution technique. The impact of the cell culture medium MEM without NE and MEM with 50 µM NE on the growth of AIEC of the density (2.5 x 10^6^ CFU/ml) corresponding to the number of AIEC used in the adherence assay was assessed spectrophotometrically at OD = 600 nm after 3 h incubation period.

### Fluorometric analysis of CEACAM6 molecule expression

The impact of NE on the CEACAM6 expression was assessed in Caco-2 cells pre-treated with NE and infected with AIEC strains as described above. After 3 h of incubation, cells were washed three times in PBS and detached using non-enzymatic cell dissociation solution, following washing three times in ice-cold PBS with 1% bovine serum albumin (PBS-BSA) and kept on ice. Then, cells were stained with mouse anti-human CEACAM6/CD66 allophycocyanin (APC)-conjugated antibody (R&D, FAB3934A). Incubation of Caco-2 cells with APC-CEACAM antibody proceeded for 45 min at 4°C, after which cells were washed three times in ice-cold PBS-BSA and diluted to the final density 3 × 10^6^ cells per well of a black-walled microtiter plate. The fluorescence was read using a Tecan Infinite M200 plate reader and nm) at the excitation wavelength of 630 nm (λex = 630 nm) and emission wavelength 665 nm (λem = 665 nm). Mouse IgG1 APC-conjugated antibody (ThermoFisher Scientific) was used as an isotype control, and uninfected cells served as a negative control. Caco-2 cells viability was determined using staining with 10 µg/mL propidium iodide (Sigma Aldrich) for 1 min, and fluorescence was measured at λem = 535 nm and λem = 617 nm. Caco-2 cells killed with 4% formaldehyde for 30 min served as a positive control in the viability assay. The assay was repeated three times in quadruplicate, and the results are presented as the mean fluorescence intensity with standard deviation.

### Yeast agglutination assay

Expression of type 1 fimbria in AIEC growing in culture conditions corresponding to adherence assay was assessed by their ability to agglutinate yeast (*Saccharomyces cerevisiae*) cells. The cultures of AIEC in SAPI-serum, MEM, and MEM-NE media were harvested and diluted with PBS to obtain a density of 9 × 10^8^ CFU/ml (established spectrophotometrically). Next, they were mixed with 1% yeast cell suspension in PBS in a 96-well microtiter plate. The agglutination reaction was inspected under an inverted microscope and recorded as the highest dilution of AIEC suspension producing yeast cell clumping. The mannose-sensitive nature of yeast agglutination was investigated in the presence of 1% methyl-α-mannopyranoside. The assay was repeated three times with three independent bacterial cultures, and the results are presented as the mean titer with standard deviation.

## Results

### Norepinephrine enhanced adherence and invasion of AIEC strains to NE-treated Caco-2 cells

**Figure 1.**
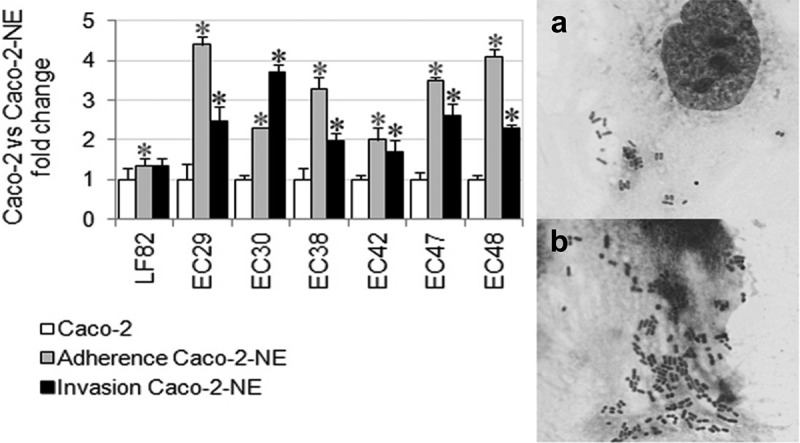
Mean adherence and invasion levels of AIEC strains to NE-treated Caco-2 cells relative to untreated Caco-2 cells set as 1. P values were determined by Student’s two-tail t-test, *, p≤0.05. Data are the means from three independent experiments performed in duplicate ± SD.The left panel presents adherence of representative AIEC strain EC48 to Caco-2 cells untreated (A) and treated with 50 µM NE (B). Wright-Giemsa staining. Magnification 100 x

The impact of NE on the interaction of AIEC with intestinal epithelial cells was assessed in an in-vitro adhesion and invasion assays to Caco-2 cell monolayers in the presence of NE (50 µM). All AIEC strains demonstrated a significant increase in adhesion (from 2- to >4-fold; p < 0.05) to NE-treated Caco-2 cells compared to untreated cells. The mean adherence level of all AIEC strains to NE-treated Caco-2 cells was significantly higher than untreated cells (20,7 x 107 CFU vs. 8.3 × 10^7^ CFU, respectively; p = 0.003). Besides, NE enhanced significantly (from 2- to 3.8-fold; p < 0.05) internalization of all wild-type AIEC by Caco-2 cells. However, the invasion of the prototype AIEC strain LF82 into NE-treated Caco-2 epithelial cells remained unchanged (p = 0.41) compared to untreated epithelial cells (Figure 1). A meta-analysis (systematic review and synthesis) has been carried out to quantify the effectiveness of NE between MEM and MEM-NE cultures of AIEC. Statistically, significant mean differences were observed in adherence (p < 0.0001) and invasion (p = 0.0001) assays – on average, higher values were in MEM-NE than in MEM medium. The graphical results are presented in forest plots (Supplementary Materials Figure S1).

#### AIEC growth in the presence of norepinephrine

**Figure 2.**
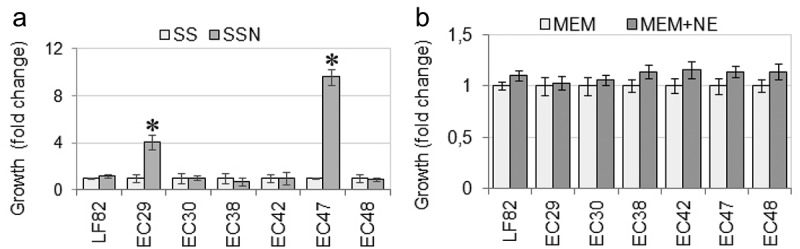
Growth of AIEC strains in SAPI-serum medium (SS) and SAPI-serum medium (SSN) supplemented with 50 mM NE (A). CFU, colony-forming units. Data were analyzed by t-test (* p<0.05) and are expressed as the means ±SD. Growth of AIEC in MEM and MEM supplemented with 50 mM NE after a 3-h incubation period estimated by measuring the optical densities of cultures at 600 nm (B). Data are the means ±SD

Supplementation of SAPI medium with bovine serum enhanced proliferation of all AIEC isolates from 10^2^ CFU/ml to 10^8^ CFU/ml after 24 hours of culture. A 24-hour exposure of AIEC to 50 μM NE significantly increased the growth of three AIEC strains (LF82, EC29, and EC47) compared to the non-supplemented medium. In contrast, NE inhibited the growth of the EC48 isolate and did not affect EC30, EC38, and EC42 strains (Figure 2). These results have indicated that the impact of NE on AIEC growth in SAPI-serum medium was a strain-dependent. On the contrary, 3-h incubation in the MEM-NE medium insignificantly enhanced the multiplication of all AIEC strains, excluding the medium’s impact and the incubation period of AIEC strains with Caco-2 cells on their increased adherence.

#### *Norepinephrine modified the expression of* the fimA *gene in AIEC strains*

The correlation between type 1 fimbria and NE-induced increased AIEC adherence and invasion of Caco-2 cells was assessed by *fimA* expression using a qRT-PCR reaction. According to culture conditions of adherence assay, RNAs were isolated from AIEC isolates cultured for 24 hours in SAPI-serum medium following subculture for 3 hours in MEM and MEM-NE (50 µM) media. The mRNA-expression levels independent of growth conditions were evaluated by the expression of *dnaE* and *rpoS* housekeeping genes in AIEC strains. The *dnaE* gene had constant expression levels for all test conditions, and the data were analyzed according to this gene (Supplementary Materials Figure S2).

**Figure 3.**
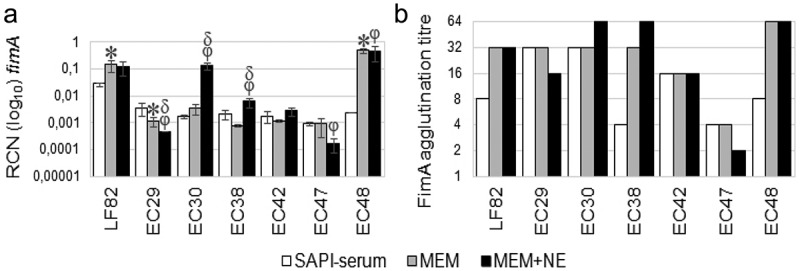
Expression of the fimA gene in AIEC strains cultured for 24 h in SAPI-serum medium and 3 h in MEM and MEM supplemented with 50 mM NE (MEM-NE). Star (*) indicates the statistical difference (p<0.05) between fimA expression in AIEC cultured in SAPI-serum and MEM media, (f) symbol indicates statistical difference (p<0.05) between fimA expression in AIEC cultured in SAPI-serum and MEM-NE media, and (d) symbol indicates the statistical difference (p<0.05) between fimA expression in AIEC cultured in MEM and MEM-NE media. Data are the means ± SD of three independent experiments in triplicate ± SD. P values were determined by Student’s two-tail t-test (A). The means of agglutination titres for AIEC strains cultured in SAPI-serum, MEM, and MEM-NE media (B)

A 3-h subculture of AIEC strains in a nutrient-rich MEM medium significantly increased mRNA *fimA* level in LF82 and EC48 strains compared to cultures in SAPI-serum medium but did not affect the gene expression in four other isolates (EC30, EC38, EC42, and EC47). However, these cultural conditions decreased *fimA* expression in EC29 isolate, indicating that a switch from a poor to a nutrient-rich environment could modulate the *fimA* gene expression in AIEC strains (Figure 3(a)). When comparing *fimA* mRNA levels in strains cultured first in SAPI-serum medium and then sub-cultured for 3-h in MEM-NE and MEM media, two isolates (EC30 and EC38) had increased expression of this gene and two strains (EC29 and EC47) had decreased expression. The remaining three isolates (LF82, EC42, and EC48) demonstrated an unchanged gene expression. Yeast agglutination assay confirmed the impact of NE on increased FimA synthesis in EC30 and EC38 strains, although the difference between MEM and MEM-NE cultures varied by only one dilution (Figure 3(b)). Spearman’s correlation analysis showed that the *fimA* gene expression in AIEC cultured in the SAPI-serum medium correlated with the adherence of AIEC to NE-treated Caco-2 cells (r = 0.6035; p = 0.02). Similarly, the *fimA* gene expression in AIEC strains cultured in SAPI-serum and then in MEM-NE correlated with the level of adhesion of tested strains to NE-treated Caco-2 cells (r = 0.5325; p = 0.04). Likewise, the *fimA* expression in strains cultured in SAPI-serum medium correlated with their invasion level to NE-treated Caco-2 cells (r = 0.5885; p = 0.02) like the *fimA* expression in strains cultured for 24 hours in SAPI-serum and then for 3 hours in MEM-NE correlated with AIEC invasion level to NE-treated Caco-2 cells (r = 0.5836; p = 0.02).

That implied that type 1 fimbria expression in AIEC strains cultured overnight in a nutrient-poor SAPI-serum medium and AIEC subcultured in the MEM-NE medium ensured their increased adhesion and invasion to NE-treated but not to untreated Caco-2 cells. On the other hand, a meta-analysis excluded the impact of NE on fimA gene expression in the presence of NE (Supplementary Materials Figure 4). This result suggested that NE may exert an effect on Caco-2 cells.

**Figure 4.**
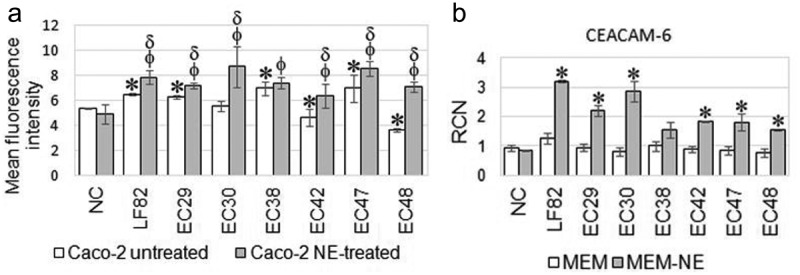
Fluorescence intensity of APC-labeled anti-hCEACM6 antibody in untreated and NE-treated Caco-2 cells infected with AIEC strains (A). Star (*) indicates the statistical difference between untreated Caco-2 cells uninfected and infected with AIEC, (d) symbol indicates the statistical difference (p<0.05) between NE-treated uninfected and infected with AIEC epithelial cells, and (f) symbol indicates statistical difference (p<0.05)  between untreated and NE-treated Caco-2 cells infected with AIEC (A). The expression of CEACAM6 gene in untreated and NE-treated Caco-2 cells infected with AIEC strains (B). P values were determined by Student’s two-tail t-test, *, p≤0.05. Data are the means ± SD of three independent experiments in triplicate ± SD

### Norepinephrine enhanced CEACA6 expression in epithelial cells infected with AIEC

The impact of NE on CEACAM6 expression in untreated and NE-treated Caco-2 cells infected with AIEC was determined with an APC-conjugated anti-hCEACAM6 antibody. Caco-2 cell viability estimated with propidium iodide was 99.7% ±0.4%. Infection of untreated Caco-2 cells with four AIEC strains LF82, EC29, EC38, and EC47 increased CEACAM6 levels compared to control uninfected Caco-2 cells. On the contrary, the CEACAM6 level in untreated Caco-2 cells infected with EC42 and EC48 strains decreased significantly (p < 0.05) compared to control cells. The infection of Caco-2 cells with EC30 isolate has not changed the CEACAM6 level comparing to untreated cells. However, the level of CEACAM6 significantly increased in NE-treated Caco-2 cells infected with all AIEC strains examined compared to untreated infected Caco-2 cells and uninfected NE-treated cells (Figure 4(a)). These results indicated that NE had a prominent effect on the level of CEACAM6 in Caco-2 cells infected with AIEC.

To established whether changes in CEACAM6 expression are related to the expression of the corresponding gene, the CEACAM6 mRNA level in NE-treated and untreated Caco-2 infected with AIEC strains was determined. The mRNA-expression levels independent of growth conditions were evaluated by the expression of *Rps-11, α-tubulin*, and *β-actin* housekeeping genes in Caco-2 cells. The *Rps-11* gene had constant expression levels for all test conditions, and the data were analyzed according to this gene (Supplementary Materials Figure S3). The result revealed that NE significantly increased CEACAM6 expression in Caco-2 cells infected with all but one AIEC strains compared to untreated cells (Figure 4(b)). The increased level of CEACAM6 mRNA in Caco-2 cells infected with EC38 strain did not reach statistical significance compared to untreated cells. The CEACAM6 mRNA level in control uninfected NE-treated cells Caco-2 cells was comparable to untreated cells. A meta-analysis confirmed the increased CEACAM6 expression in NE-treated Caco-2 cells compared to untreated cells infected with AIEC (Supplementary Materials Figure S5). The Pearson correlation coefficient of 0.912 indicated a strong positive correlation between *ceacam6* gene expression and CEACAM6 synthesis in NE-treated Caco-2 cells infected with AIEC.

## Discussion

Lyte [21] and Freestone [22] demonstrated that catecholamine hormones increase virulence genes’ expression, vital to bacterial ability to interact with host cells. In the study, we investigated the effect of NE on the adherence and invasion of six wild-type AIEC strains isolated from children with Crohn’s disease to Caco-2 cells. An in vitro assay showed that NE significantly increased the adherence and invasion of AIEC to intestinal epithelial cells. Considering that NE increases the proliferation of many bacteria species, we first explored the effect of NE on the multiplication of AIEC in a nutrient-poor SAPI-serum medium. We found that this hormone increased AIEC proliferation. However, the influence was strain-dependent, which excluded the impact of increased AIEC proliferation on enhanced adherence and invasion into intestinal epithelial cells for most AIEC strains studied. Similarly, although the AIEC population’s density in a nutrient-rich MEM medium has increased in the presence of NE, it was not significant, excluding the effect of short-term culture in a nutrient-rich medium on NE-induced increased AIEC adhesion and invasion.

The interaction of AIEC with intestinal epithelial cells requires type 1 fimbriae. According to Boudeau et al. [4] AIEC present variant of type 1 pili that provide the adherence and invasion of AIEC into epithelial cells. In the study, the *fimA* gene expression required for type 1 piliation was assessed after three-hours of incubation in the cell culture medium supplemented with NE and compared to the gene expression in AIEC cultured SAPI-serum and MEM media without the hormone according to the adherence assay conditions. The results indicated that the hormone had a strain-dependent effect on *fimA* expression in AIEC that did not reflect increased adherence of all strains assessed. Moreover, meta-analysis excluded the impact of NE on *fimA* gene expression in AIEC and enhanced adherence of AIEC to epithelial cells.

In addition to bacterial factors, the epithelial cells’ receptors play a crucial role in the interaction of the intestinal pathogens with epithelial host cells. CEACAM6 is a cell adhesion receptor of the immunoglobulin-like superfamily anchored to the cell membrane. CEACAM6 regulates cell adhesion, proliferation, signaling in cancer, and immunity [23]. Barnich et al. [4] demonstrated that CEACAM6 acts as a receptor for AIEC adherence, and its expression enhanced in cultured epithelial cells after infection with AIEC bacteria. Moreover, according to Denizot et al. [24], CEACAM6 is overexpressed in patients with CD favoring AIEC colonization. The overexpression of CEACAM6 combined with AIEC infection leads to abnormal intestinal permeability and disruption of intestinal epithelial barrier function accompanied by translocation of AIEC via intestinal epithelium and cytokine secretion.

Considering the importance of CEACAM6 for the interaction of AIEC with intestinal epithelial cells, in the study, we investigated the effect of NE on the expression of CEACAM6 in Caco-2 cells infected with AIEC. The result indicated that NE had no impact on the CEACAM6 level in control, uninfected cells. On the other hand, NE significantly increased the expression of CEACAM6 in AIEC-infected Caco-2 cells compared to untreated but infected cells. Barnich et al. [4] have demonstrated that AIEC strains indirectly up-regulated CEACAM6 in epithelial cells via activation of proinflammatory cytokine tumor necrosis factor-alpha (TNFα). Whether NE increases TNFα synthesis and secretion or modulates the CEACAM6 expression via other mechanisms is under investigation in our laboratory. In normal physiological conditions, NE as an agonist of α- and β-adrenergic receptors, reduced TNFα expression in monocytes challenged with LPS, implying its anti-inflammatory role in bacterial infections [25]. The study of Schmidt et al. [26] confirmed the impact of NE as an agonist of adrenergic receptors on intestinal mucosal defense via rapid secretory IgA secretion in mucosal explants from the porcine distal colon. In turn, Spencer et al. [27] demonstrated that in *S*. Typhimurium, NE downregulated the virulence gene expression that affects their survival and long-term persistence host. Moreover, they also showed that NE increased the sensitivity of *S*. Typhimurium to LL-37 antibacterial peptide. These data imply that catecholate hormones may exert a dual effect on pathogenic bacteria by promoting their virulence and growth in the host from one side and increasing their sensitivity to an innate immune response from the other side. Hence, the outcome of infection with intestinal pathogens may depend on the NE-amendable delicate balance between innate immune response and bacterial virulence.

The study results have shown that NE, similarly to TNFα, increased CEACAM6 expression in intestinal epithelial cells infected with AIEC. Considering the role of CEACAM6 in many different cellular processes, the ability of NE to increase its level in AIEC infected epithelial cells, the hormone, maybe a relevant factor in the pathomechanism of CD disease.

## Conclusions

The results of the study indicated that NE increased the adhesion and invasion of AIEC to Caco-2 cells and modulated the *fimA* gene expression essential for the interaction of AIEC with intestinal epithelial cells. Notably, norepinephrine enhanced CEACAM6 expression in intestinal epithelial cells infected with AIEC, implying that the hormone can facilitate AIEC colonization of intestinal epithelium by affecting the expression of cellular receptors.

## Supplementary Material

Supplemental Material
