# Supplementary material for: Norepinephrine affects the interaction of adherent-invasive Escherichia coli with intestinal epithelial cells
Source: Virulence. 2021 Feb 4;12(1):630–7. doi: 10.1080/21505594.2021.1882780 (PMC7872043; doi:10.1080/21505594.2021.1882780)
Supplement: Supplemental Material [file KVIR_A_1882780_SM5359.docx]

**Supplementary materials**

*E. coli* strains isolated from biopsy specimens were classified as AIEC pathotype on the basis of adherence and invasion assays, and the ability to survive in macrophages (Table 1).

**Table S1.** Characteristics of *E. coli* strains examined in the study.

| *E. coli* strain | Adherence  Int407  (x10^7^ CFU/mL)^1^ | Invasion Int407 (%)^1^ | Survival in macrophages^2^ (%) | Phylogroup^3^ |
| --- | --- | --- | --- | --- |
| EC29 | 7.8±0.9 | 12.9±1.5 | 978.7±147.3 | B2 |
| EC30 | 14±5 | 1.1±0.3 | 407.2±58.7 | B2 |
| EC38 | 4.5±0.5 | 3.7±1.1 | 119.8±11 | D |
| EC42 | 6.8±1.3 | 1.5±0.6 | 102.5±33.7 | B2 |
| EC47 | 17±4 | 1.6±0.5 | 403.3±11.5 | B2 |
| EC48 | 19.7±6.3 | 0.4±0.07 | 420±99.5 | B2 |
| LF82 | 11.5±1.4 | 2.3±0.8 | 37,2±8.6 | B2 |

^1^ **Adherence and invasion assays to Int407 cell line**. Overnight *E. coli* cultures in Luria broth medium were harvested, resuspended in saline to OD=6 x10^8^ CFU/ml and used to infect Int407 cells at a multiplicity of infection (MOI) of 50 bacteria per cell. A 3-h postinfection, cells were washed three times and lysed with 0.1% Triton X-100. Serial dilutions of bacterial lysates were plated onto nutrient agar and incubated overnight at 37° C to count bacterial colonies (CFU). The gentamicin-protection internalization assay was performed in the same manner as the adherence assay with an additional 1 h of incubation of Int407 cells in gentamicin-containing MEM (100 µg/mL) medium in order to kill adherent bacteria. Three separate experiments for adherence and invasion assays were performed in triplicate. Data are the mean from three independent experiments ± standard deviation.

^2^ **Phagocytosis assay.** The ability of *E. coli* to survive in macrophages was examined in the THP-1 human monocyte-like cell line (ATTC TIB-202). THP-1 human monocyte-like cell line cells were routinely cultured in RPMI 1640 medium supplemented with 10% FBS, 2 mM glutamine and 100 U/ml penicillin and 100 µg/ml streptomycin at 37°C in an atmosphere with 5% CO_2_. THP-1 cells (5 x 10^5^ cells per well) were seeded on a 24-well plate and differentiated into monocyte-derived macrophages for 48 h with 25 nM phorbol 12-myristate 13-acetate (PMA). After differentiation THP-1 cells were left to rest for an additional 48 h prior infection. Then, after thorough washing with pre-warmed PBS supplemented with 2% FBS, a fresh culture medium without antibiotic was added and the cells were incubated an additional 24 h prior to infection. MDM were infected with *E. coli* strains cultured for 24 h in Luria broth medium at MOI of 10 and centrifuged at 180 x g for 10 min to synchronize phagocytosis. Infected MDM were incubated for an additional 20 min at 37°C in an atmosphere with 5% CO_2_. After incubation, the cell culture medium was replaced by RPMI 1640 with 100 µg/ml gentamycin and incubated for 1 h to kill extracellular bacteria. Then, macrophages were washed twice with pre-warmed PBS supplemented with 2% FBS and lysed with 1% Triton X-100 in deionized water to release intracellular bacteria or maintained for 24 h in RPMI 1640 with 10% FBS and 20 µg/ml gentamycin to continually exclude all intracellular bacteria. Cellular lysates were serially diluted in PBS and plated onto MacConkey agar to estimate colony forming units (CFU). Survival was expressed as the mean percentage of the number of *E. coli* recovered after 30 min postinfection, defined as 100%. Data are the mean from three independent experiments ± standard deviation.

^3^ **Phylogroups** were determined according to Clermont et al. [1]. PCR amplifications were performed in a DNA-Engine PT200 thermal cycler (MJ Research Waltham, MA, USA). The PCR products were visualized after electrophoresis on 2% agarose gel in Tris-acetate-EDTA buffer by staining with SYBR Green I Nucleic Acid Gel Stain.

**Table S2**. Primers used in the study in PCR and RT-qPCR reactions.

| Gene | Primer sequence (5’-3’) | Amplicon size (bp) | Reference of source |
| --- | --- | --- | --- |
| Primers used in **PCR reaction** | | | |
| *fimA* | TGCAGAACGGATAGGCCGTGG  GCAGTCACCTGCCCTCCGGTA | 508 | [2] |
| Primers used in **RT-qPCR** | | | |
| *fimA*  *rpoS*  *dnaE*  *CEACAM6*  *Rps-11*  *α-tubulin*  *β-actin* | GCTGAGCCTGGGTGTTTCCT  TCCAGAGCAGCCTGACCTTC  ATGTCGGAGGCGTAAGGCT  TCCAGGGCGTCAGTAAACAA  TGCAAGTCGAACGGTAACAG  AGTTATCCCCCTCCATCAGG  GCCGAGACTATCTGCACTAC  ATGTCCAGCCTCAGAACTTC  GCCTGGACCACAAGTTTGAC  TGAAATTCTGGGAGCATGAC  ACTGGAACGGTGAAGGTGAC  GTGGACTTGGGAGAGGACTG  GCCTGGACCACAACTTTGAC  TGAAATTCTGGGAGCATGAC | 134  183  181  179  222  236  204 | [3]  [4]  [5]  [6]  [6]  [6]  [6] |

**Figure S1.** Metaanalysis of the adherence (A) and invasion (B) of AIEC to untreated and NE-treated Caco-2 cells. To combine quantitative results from both, adherence and invasion, meta-analysis (systematic review and synthesis) has been carried out to quantify the effectiveness of NE between MEM and MEM-NE cultures. A meta-analysis model that allows appropriate and efficient modeling of data from comparative studies reporting formats on the mean difference scale was used. Since a test for heterogeneity for all assays was significant (p<0.0001), a random-effect model was applied according to Sutton et al. [7]. The meta-analysis indicated that statistically significant mean differences were observed in adherence and invasion – on average, higher values were in MEM-NE than in MEM medium. The metaanalysis confirmed that NE enhanced AIEC adherence and invasion into intestinal epithelial cells.


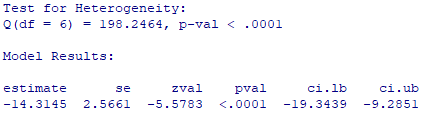


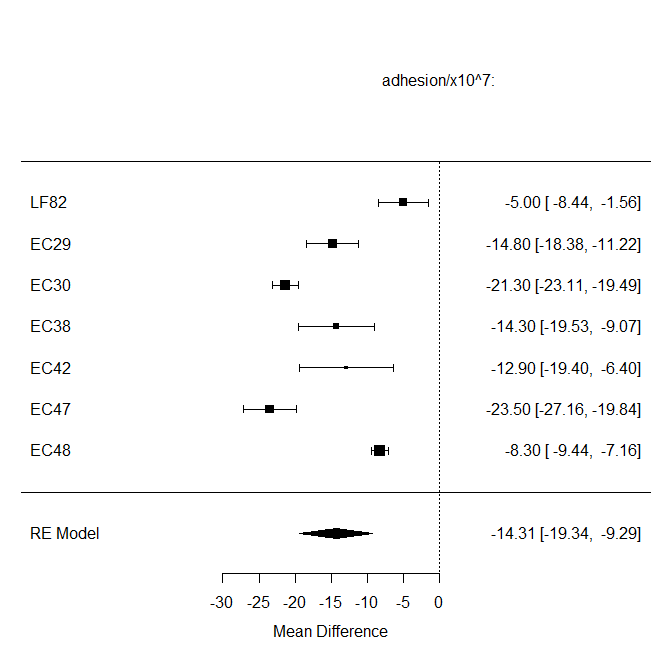


A


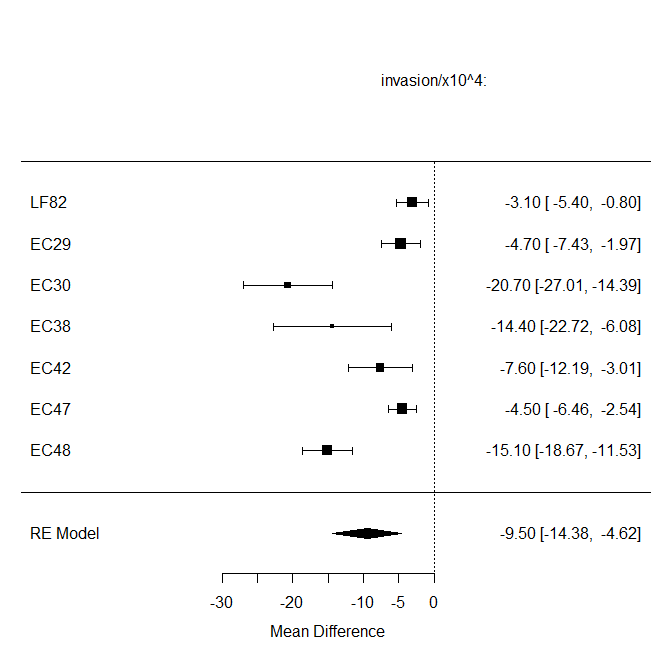


B


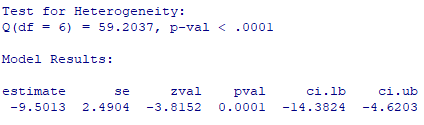


**Figure S2.** The standard deviations of the expression of dnaE and rpoS housekeeping genes in AIEC strains cultured for 24 h in SAPI-serum medium (SS) following 3 h incubation in MEM medium or in MEM medium supplemented with 50 µM NE.

The dnaE gene had constant expression levels for all test conditions, and the data were analyzed according to this gene.


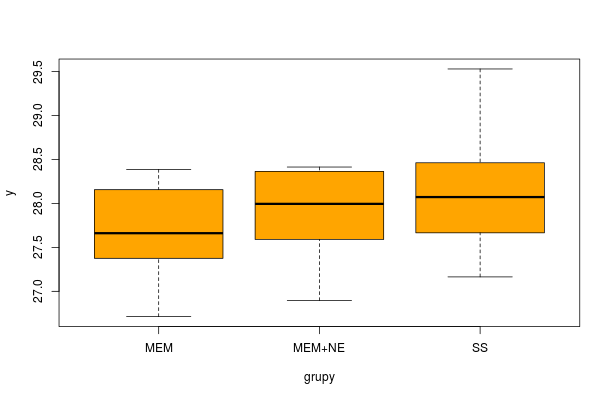


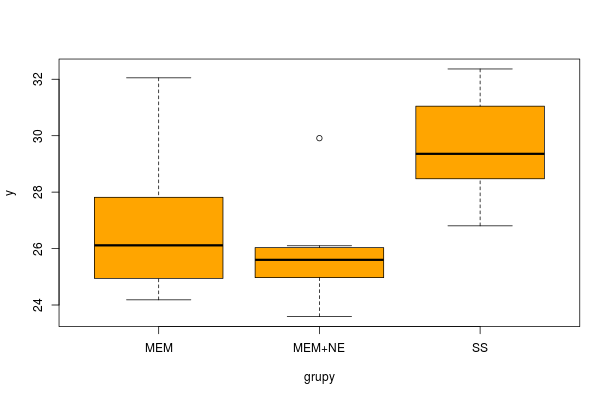


**Figure S3.** Standard deviations of housekeeping genes for analysis of CEACAM6 mRNA in Caco-2 cells under experiments conditions.

The Rps-11 gene demonstrated the lowest standard deviations in the experiment conditions and the data were analyzed according to this gene.

**Figure S4.** The impact of NE on the *fimA* gene expression in AIEC in the presence and absence of norepinephrine. The p values = 0.6113 and 0.3095 excluded correlation between *fimA* gene expression in AIEC cultured in the presence of NE as well as between *fimA* expression in the presence of NE and adhesion level..


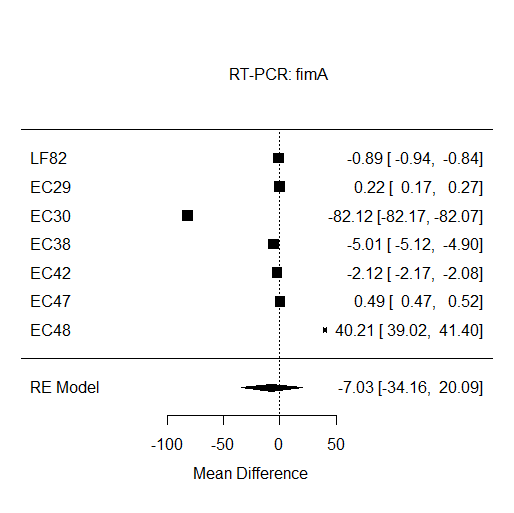


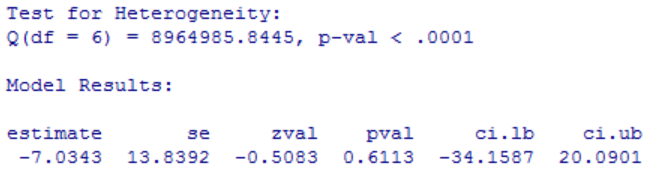


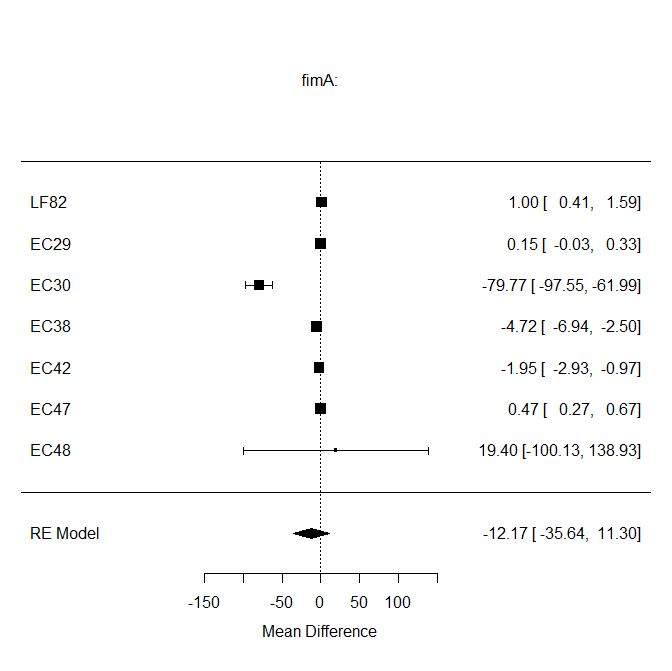


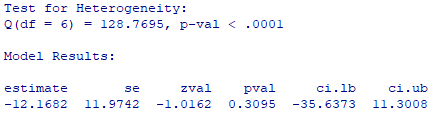


**Figure S5.** Dependency of CEACAM6 molecule expression on the surface of Caco-2 cells on increased level of CEACAM6 mRNA in Caco-2 cells infected with AIEC strains.


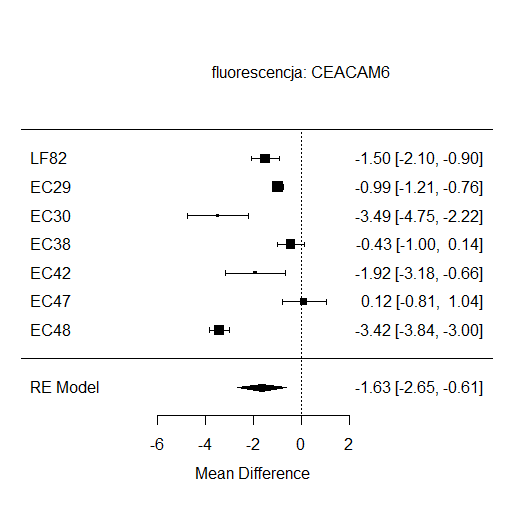


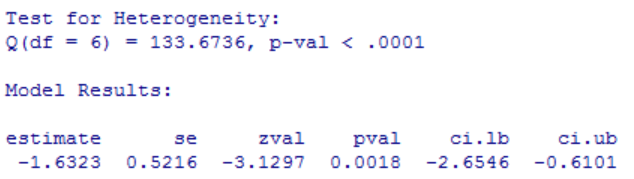


**References**

1. **Clermont** O, Bonacrosi S, Bingen E. Rapid and simple determination of the *Escherichia coli* phylogenetic groups. Appl Environ Microbiol 2000, 66, 4555–4558. PMID: [11010916](https://www.ncbi.nlm.nih.gov/pubmed/11010916)
2. **Johnson** JR, Stell AL. Extended virulence genotypes of *Escherichia coli* strains from patients with urosepsis in relation to phylogeny and host compromise. J. Infect Dis 2000; 181: 261-271. DOI:[10.1086/315217](https://doi.org/10.1086/315217)
3. **Sharma** VK, Bearson SM, Bearson BL. Evaluation of the effects of *sdiA*, a *luxR* homologue, on adherence and motility of *Escherichia coli* O157:H7. Microbiol. 2010;156:1303-12. DOI:[10.1099/mic.0.034330-0](https://doi.org/10.1099/mic.0.034330-0)
4. **Lloyd** SJ, Ritchie JM, Rojas-Lopez M, Blumentritt CA, Popov VL, Greenwich JL, Waldor MK, Torres AG. A double, long polar fimbria mutant of *Escherichia coli* O157:H7 expresses curli and exhibits reduced *in vivo* colonization. Infect Immun 2012; 80: 914-920. doi: [10.1128/IAI.05945-11](https://dx.doi.org/10.1128%2FIAI.05945-11)
5. **Wang** S, Niu C, Shi Z, Xia Y, Yaqoob M, Dai J, Lu C. Effects of *ibeA* deletion on virulence and biofilm formation of avian pathogenic *Escherichia coli*. Infect Immun 2011; 79: 279-287. DOI: [10.1128/IAI.00821-10](https://doi.org/10.1128/IAI.00821-10)
6. Roda G, Dahan S, Mezzanotte L, Caponi A, Roth-Walter F, Pinn D, Mayer L. The defect in CEACAM family member expression in Crohn’s disease IECs is regulated by the transcription factor SOX9. Inflamm Bowel Ds. 2009;15:1775-1783. doi: [10.1002/ibd.21023](https://dx.doi.org/10.1002%2Fibd.21023)
7. **Sutton** A., Abrams K., Jones D. An illustrated guide to the methods of meta-analysis. Journal of Evaluation in Clinical Practice 2001;7,2()135-148.
